# Supplementary material for: Modularity and determinants of a (bi-)polarization control system from free-living and obligate intracellular bacteria
Source: eLife. 2016 Dec 23;5:e20640. doi: 10.7554/eLife.20640 (PMC5182065; doi:10.7554/eLife.20640)
Supplement: Supplementary file 1. — Data used to derive the NMR solution structure. DOI: http://dx.doi.org/10.7554/eLife.20640.016 [file elife-20640-supp1.docx]

**- NMR and refinement statistics of ZitP^(1-43)^.**

|  | **ZitP^(1-43)^** |
| --- | --- |
| **NMR distance and dihedral constraints** |  |
| Distance constraints |  |
| Total NOE | 440 |
| Intra-residue | 135 |
| Inter-residue | 305 |
| Sequential (\|*i* – *j*\| = 1) | 131 |
| Medium-range (\|*i* – *j*\| < 4) | 53 |
| Long-range (\|*i* – *j*\| > 5) | 101 |
| Hydrogen bonds | 10 |
| Total dihedral angle restraints | 62 |
| φ | 31 |
| ψ | 31 |
|  |  |
| **Structure statistics** |  |
| Violations (mean and s.d.) |  |
| Distance constraints > 0.2Å | 3.5 +/- 1.10 |
| Dihedral angle constraints >5º | 0.0 +/- 0.00 |
| Max. dihedral angle violation (º) | 0.70 +/- 0.13 |
| Max. distance constraint violation (Å) | 0.28 +/- 0.01 |
| Deviations from idealized geometry |  |
| Bond lengths (Å) | 0.0043 |
| Bond angles (º) | 1.083 |
| Average pairwise r.m.s. deviation** (Å) |  |
| Backbone | 0.23 +/- 0.09 |
| Heavy atoms | 0.76 +/- 0.11 |

** Statistics computed for the deposited bundle of 20 NMR structures selected

based on residue range 2-38, see accession numbers 2NB9 (PDB database) and 25967 (BMRB database).
