# Supplementary material for: Modularity and determinants of a (bi-)polarization control system from free-living and obligate intracellular bacteria
Source: eLife. 2016 Dec 23;5:e20640. doi: 10.7554/eLife.20640 (PMC5182065; doi:10.7554/eLife.20640)
Supplement: Supplementary file 2. — Details on strain and plasmid constructions and the oligonucleotides that were used. DOI: http://dx.doi.org/10.7554/eLife.20640.017 [file elife-20640-supp2.docx]

***C. crescentus* and *E. coli* strains**

| Name | Relevant genotype/ description | Construction, source or ref |
| --- | --- | --- |
| ***C. crescentus* strains** | | |
| NA1000 | Synchronizable derivative of wild-type strain CB15 | Evinger and Agabian, 1977 |
| CAG9 | NA1000; *zitP::Tn5-GFP-* | Hughes et al., 2010 |
| UG2202 | NA1000; *parB*::*GFP*-*parB* | Thanbichler and Shapiro, 2006 |
| LT1050  (JP408) | NA1000; *parB::CFP-parB; popZ::mCherry-popZ* | Ptacin et al., 2014 |
| LT1051  (JP502) | NA1000; *parB*::*CFP-parB; popZ:: mCherry -popZ^E12K/R19E^* | Ptacin et al., 2014 |
| LT1052  (JP470) | NA1000; *parB*::*CFP-parB; popZ:: mCherry -popZ^S22P^* | Ptacin et al., 2014 |
| LT1053  (JP451) | NA1000; *parB*::*CFP-parB; popZ:: mCherry -popZ^KEP^* | Ptacin et al., 2014 |
| GB255 | NA1000; *ΔpopZ::spc* | Bowman et al., 2008 |
| MB1227 | NA1000; Δ*zitP* | Mating of pMB123 into NA1000 (double homologous recombination) |
| MB1053 | NA1000; *zitP::Tn5-GFP* | Transduction of CAG9 into NA1000 |
| MB1055 | NA1000; *zitP::Tn5-GFP; ΔpopZ::spc* | Transduction of *ΔpopZ::spc* in MB1053 |
| MB401 | NA1000; *xylX::dendra2-zitP*^1-90Cc^ | Single integration of pMB22 into NA1000 |
| MB542 | NA1000; *xylX::dendra2-zitP*^1-90Ae^ | Single integration of pMB33 into NA1000 |
| MB588 | NA1000; *xylX::dendra2- zitP*^1-90Mm^ | Single integration of pMB38 into NA1000 |
| MB590 | NA1000; *xylX::dendra2-zitP*^1-90Cs^ | Single integration of pMB39 into NA1000 |
| MB468 | NA1000; *ΔpopZ::spc; xylX::dendra2-zitP*^1-90Cc^ | Transduction of *ΔpopZ::spc* in MB401 |
| MB706 | NA1000; *ΔpopZ::spc; xylX::dendra2-zitP*^1-90Ae^ | Transduction of *ΔpopZ::spc* in MB542 |
| MB708 | NA1000; *ΔpopZ::spc; xylX::dendra2-zitP*^1-90Mm^ | Transduction of *ΔpopZ::spc* in MB588 |
| MB710 | NA1000; *ΔpopZ::spc; xylX::dendra2-zitP*^1-90Cs^ | Transduction of *ΔpopZ::spc* in MB590 |
| MB852 | NA1000; *xylX::dendra2-zitP*^1-43Cc^ | Single integration of pMB66 into NA1000 |
| MB1252 | NA1000; *ΔzitP ; xylX::dendra2-zitP*^1-43Cc^ | Single integration of pMB66 into MB1227 |
| MB1250 | NA1000; Δ*zitP ; xylX::dendra2-zitP*^1-133Cc^ | Single integration of pMB23 into MB1227 |
| MB894 | NA1000; Δ*popZ::spc; xylX::dendra2-zitP*^1-43Cc^ | Transduction of *ΔpopZ::spc* in MB852 |
| MB856 | NA1000; *xylX*::*dendra2-zitP*^1-43Bd^ | Single integration of pMB73 into NA1000 |
| MB1093 | NA1000; *ΔpopZ::spc*; xylX::*dendra2-zitP*^1-43Bd^ | Transduction of *ΔpopZ::spc* in MB856 |
| MB1413 | NA1000; *popZ::mCherry-PopZ* | Transduction of *m****C****herry -popZ* into NA1000 |
| MB1448 | NA1000; Δ*zitP*; *popZ::mcherry-PopZ* | Transduction of *m****C****herry -popZ* into MB1227 |
| MB1707 | NA1000; Δ*zitP*; *xylX::dendra2-zitP*^1-43Cc^; *popZ::mcherry-PopZ* | Single integration of pMB66 into MB1448 |
| MB460 | NA1000; *xylX::dendra2-AgmX*^1-90^ | Single integration of pMB26 into NA1000 |
| MB1549 | NA1000; Δ*zitP*; *xylX::dendra2-zitP^1-43Cc^;* pMT335 | Transformation of pMT335 into MB1252 |
| MB1551 | NA1000; Δ*zitP*; *xylX::dendra2-zitP^1-43Cc^;* pMT335-*popZ* | Transformation of pMB21 into MB1252 |
| MB1248 | NA1000; Δ*zitP*; *xylX::dendra2-zitP* | Single integration of pXdendra-2N2-zitP into MB1227 |
| MB313 | NA1000; Δ*zitP*; *xylX::dendra2-zitP;* pMT335-*popZ* | Transformation of pMB21 into MB1248 |
| MB1731 | NA1000; *xylX*::*dendra2-zitP*^1-43CS^ | Single integration of pMB164 into NA1000 |
| MB1264 | NA1000; *xylX*::*dendra2-zitP*^1-43W35I^ | Single integration of pMB115 into NA1000 |
| MB1580 | NA1000; *xylX*::*dendra2-zitP*^1-43R24A/R27A^ | Single integration of pMB170 into NA1000 |
| MB1723 | NA1000; *xylX*::*dendra2*-*zitP*^1-43WT(Rs)^ | Single integration of pMB184 into NA1000 |
| MB1258 | NA1000; *xylX*::*dendra2*-*zitP*^1-43Q27R(Rs)^ | Single integration of pMB91 into NA1000 |

| MB1725 | NA1000; *xylX*::*dendra2*-*zitP*^1-43Q27R/W35I(Rs)^ | Single integration of pMB185 into NA1000 |
| --- | --- | --- |
| MB1668 | NA1000; Δ*popZ* ; xylX::*dendra2*-*zitP*^1-43Q27R(Rs)^ | Transduction of *ΔpopZ::spc* into MB1258 |
| MB1672 | NA1000; *parB::parB-GFP; popZ::mCherry-popZ;* pMT464*-zitP*^1-133WT^-*popZ* | Transformation of pMB175 into LT1050 |
| MB1674 | NA1000; *parB::parB-GFP; popZ::mCherry-popZ;* pMT464*-zitP*^1-133W35I^-*popZ* | Transformation of pMB177 into LT1050 |
| MB1156 | NA1000; *parB::parB-GFP; popZ::mCherry-popZ;* pMT464 | Transformation of pMT464 into LT1050 |
| MB1158 | NA1000; *parB::parB-GFP; popZ:: mCherry -popZ;* pMT464*-zitP*^1-133WT^ | Transformation of pMB93 into LT1050 |
| MB2163 | NA1000; *parB::parB-GFP; popZ:: mCherry -popZ;* pMT464*-zitP*^1-90WT^ | Transformation of pMB255 into LT1050 |
| MB2165 | NA1000; *parB::parB-GFP; popZ:: mCherry -popZ;* pMT464*-zitP* | Transformation of pMB256 into LT1050 |
| MB2167 | NA1000; *parB::parB-GFP; popZ:: mCherry -popZ;* pMT464*-zitP*^1-90^-*popZ* | Transformation of pMB257 into LT1050 |
| MB2169 | NA1000; *parB::parB-GFP; popZ:: mCherry -popZ;* pMT464*-zitP*-*popZ* | Transformation of pMB258 into LT1050 |
| MB1160 | NA1000; *parB::parB-GFP; popZ:: mCherry -popZ;* pMT464*-zitP*^1-133CS^ | Transformation of pMB94 into LT1050 |
| MB1221 | NA1000; *parB::parB-GFP; popZ:: mCherry -popZ;* pMT464*-zitP*^1-133W35I^ | Transformation of pMB113 into LT1050 |
| MB1670 | NA1000; *parB::parB-GFP; popZ:: mCherry -popZ;* pMT464*-zitP*^1-133R24A/R27A^ | Transformation of pMB171 into LT1050 |
| MB1225 | NA1000; *parB::parB-GFP; popZ:: mCherry -popZ;* pMT464*-zitP*^1-133malF^ | Transformation of pMB127 into LT1050 |
| MB2159 | NA1000; *parB::parB-GFP; popZ:: mCherry -popZ*^KEP^*;* pMT464*-zitP*^1-133^ | Transformation of pMB93 into LT1053 |
| MB2161 | NA1000; *parB::parB-GFP; popZ:: mCherry -popZ*^KEP^*;* pMT464*-zitP*^1-133W35I^ | Transformation of pMB113 into LT1053 |
| MB1501 | NA1000; *parB::parB-GFP; popZ:: mCherry -popZ;* pMT464*-zitP*^1-120Bd^ | Transformation of pMB156 into LT1050 |
| MB1707 | NA1000; Δ*zitP*; *popZ*:: *mCherry -popZ*; *xylX*::*dendra2-zitP*^1-43^ | Single integration of pMB66 into MB1448 |
| MB1709 | NA1000; Δ*zitP*; *popZ*:: *mCherry -popZ*; *xylX*::*dendra2-zitP*^1-133^ | Single integration of pMB23 into MB1448 |
| MB1424 | NA1000; *vanA*::*parA*_G16V_-*eYFP* | Transduction of *parA_G16V_-eYFP* into NA1000 |
| MB1428 | NA1000; *vanA*::*parA*_G16V_-*eYFP*; pMT464 | Transformation of pMT464 into MB1424 |
| MB1430 | NA1000; *vanA*::*parA*_G16V_-*eYFP*; pMT464-*zitP*^1-133WT^ | Transformation of pMB93 into MB1424 |
| MB1432 | NA1000; *vanA*::*parA*_G16V_-*eYFP*; pMT464-*zitP*^1-133CS^ | Transformation of pMB94 into MB1424 |
| MB1440 | NA1000; *popZ*::*mCherry*-*popZ* | Transduction of *mcherry-popZ* (from LT1050) into NA1000 |
| MB1442 | NA1000; *popZ*::*mCherry*-*popZ*^E12K/R19E^ | Transduction of *mcherry-popZ*^E12K/R19E^ (from LT1051) into NA1000 |
| MB1444 | NA1000; *popZ*:: *mCherry* -*popZ*^S22P^ | Transduction of *mcherry-popZ*^S22P^ (from LT1052) into NA1000 |
| MB1446 | NA1000; *popZ*:: *mCherry* -*popZ*^KEP^ | Transduction of *mcherry-popZ*^KEP^ (from LT1053) into NA1000 |
| MB1450 | NA1000; Δ*zitP*; *popZ*::*mCherry*-*popZ*^E12K/R19E^ | Transduction of *mcherry-popZ*^E12K/R19E^ (from LT1051) into MB1227 |
| MB1452 | NA1000; Δ*zitP*; *popZ*:: *mCherry* -*popZ*^S22P^ | Transduction of *mcherry-popZ*^S22P^ into MB1227 |
| MB1454 | NA1000; Δ*zitP*; *popZ*:: *mCherry* -*popZ*^KEP^ | Transduction of *mcherry-popZ*^KEP^ into MB1227 |
| MB1469 | NA1000; *popZ:: mCherry -popZ*^Δ26^ | Mating of pMB145 into GB255 (double homologous recombination) |
| MB1509 | NA1000; *popZ mCherry -popZ*^Δ26^ | Transduction of *mcherry-popZ*^Δ26^ into NA1000 |
| MB1511 | NA1000; Δ*zitP*; *popZ:: mCherry -popZ*^Δ26^ | Transduction of *mcherry-popZ*^Δ26^ into MB1227 |
| MB1701 | NA1000; *popZ*::*mCherry*-*popZ; xylX*::*GFP* | Transformation of pX-GFP into MB1440 |
| MB1703 | NA1000; Δ*zitP*; *popZ::mcherry-PopZ; xylX*::*GFP* | Transformation of pX-GFP into MB1448 |
| MB1711 | NA1000; *popZ*::*mCherry*-*popZ*^E12K/R19E^*; xylX*::*GFP* | Transformation of pX-GFP into MB1442 |
| MB1713 | NA1000; Δ*zitP*; *popZ*::*mCherry*-*popZ*^E12K/R19E^ *xylX*::*GFP* | Transformation of pX-GFP into MB1450 |
| MB1715 | NA1000; Δ*zitP*; *popZ*::*mCherry*-*popZ*^E12K/R19E^ *xylX*::*dendra2-zitP* | Transformation of pX-dendra2-N2-zitP into MB1450 |
| MB1717 | NA1000; Δ*zitP*; *popZ*::*mCherry*-*popZ*^E12K/R19E^ *xylX*::*dendra2-zitP*^1-43Cc^ | Transformation of pMB66 into MB1450 |
| MB1719 | NA1000; Δ*zitP*; *popZ*::*mCherry*-*popZ*^E12K/R19E^ *xylX*::*dendra2-zitP*^1-133Cc^ | Transformation of pMB23 into MB1450 |
| MB2201 | NA1000; Δ*zitP*; *popZ*::*mCherry*-*popZ*^E12K/R19E^ *xylX*::*dendra2-zitP*^1-133(CS)Cc^ | Transformation of pMB25 into MB1450 |
| MB1642 | NA1000; Δ*zitP*; *parB*::*CFP*-*parB;* *popZ:: mCherry -popZ* | Transduction of CC1227 carrying pMB123 (double homologous recombination) into LT1050 |
| MB1647 | NA1000; Δ*zitP*; *parB*::*CFP*-*parB;* *popZ:: mCherry -popZ*^E12K/R19E^ | Transduction of CC1227 carrying pMB123 (double homologous recombination) into LT1051 |
| MB1016 | NA1000; *parB*::*GFP-parB*; pMT464-*zitP*^1-133WT^ | Transformation of pMB93 into UG2202 |
| MB1018 | NA1000; *parB*::*GFP-parB*; pMT464-*zitP*^1-133CS^ | Transformation of pMB94 into UG2202 |
| MB1625 | NA1000; *popZ:: mCherry -popZ*^Δ26^; *xylX*::*dendra2*-*zitP* | Single integration of pX-dendra2-2N2-*zitP* into MB1509 |
|  |  |  |
|  |  |  |

| MB1627 | NA1000; *popZ:: mCherry -popZ*^Δ26^; *xylX*::*dendra2*-*zitP*^1-43^ | Single integration of pMB66 into MB1509 |
| --- | --- | --- |
| MB1629 | **NA1000; *popZ:: mCherry -popZ*^Δ26^; *xylX*::*dendra2*-*zitP*^1-133^** | Single integration of pMB23 into MB1509 |
| MB929 | NA1000; *xylX*::*dendra2*-*zitP*^1-43WT(Rm)^ | Single integration of pMB81 into NA1000 |
| MB1266 | NA1000; *xylX*::*dendra2*-*zitP*^1-43W35I(Rm)^ | Single integration of pMB116 into NA1000 |
| MB1095 | NA1000; Δ*popZ::spc*; *xylX*::*dendra2*-*zitP*^1-43WT(Rm)^ | Transduction of *ΔpopZ::spc* into MB929 |
| MB1223 | NA1000; *parB::CFP*-parB*; popZ:: mCherry -popZ;* pMT464*-zitP*^1-104WT(Rm)^ | Transformation of pMB114 into LT1050 |
| MB2217 | NA1000; *parB::parB-GFP; popZ:: mCherry -popZ;* pMT464*-zitP*^1-104(Rm)^-*popZ* | Transformation of pMB265 into LT1050 |
| ***E. coli* strains** |  |  |
| S17-1 | RP4,Tc::Mu Km::Tn7 | Simon et al., 1983 |
| EC100D | F-mcrA Δ(mrr-hsdRMS-mcrBC) Φ80dlacZΔM15 ΔlacX74 recA1 endA1 araD139 Δ(ara, leu)7697 galU galK λ-rpsL (StrR) nupG | Epicentre |
| MG1655 | WT E. coli | Guyer et al., 1981 |
| TB28 | MG1655; Δ*lacIZYA* | Bernhardt and De Boer, 2003 |
| eMB255 | S17-1; pNTPS138-Δ*zitP* | Transformation of pMB123 into S17-1 |
| eMB283 | S17-1; pMCS4- *mCherry -popZ*^Δ26^ | Transformation of pMB145 into S17-1 |
| eMB119 | MG1655; pSRK-*dendra2*-*zitP* | Transformation of pMB43 into MG1655 |
| eMB121 | MG1655; pSRK-*dendra2*-*zitP*^1-90WT(Cc)^ | Transformation of pMB45 into MG1655 |
| eMB325 | TB28; pSRK-*dendra2*-*zitP*^1-133wt(Cc)^ | Transformation of pMB165 into TB28 |
| eMB286 | TB28; pBAD101 | Transformation of pBAD101 into TB28 |
| eMB288 | TB28; pBAD101- *mCherry* -*popZ* | Transformation of pMB59 into TB28 |
| eMB290 | TB28; pBAD101- *mCherry* -*popZ*^Δ26^ | Transformation of pMB128 into TB28 |
| eMB334 | TB28; pBAD101- *mCherry* -*popZ*; pSRK-*dendra2*-*zitP*^1-43WT^ | Transformation of pMB165 into eMB288 |
| eMB336 | TB28; pBAD101- *mCherry -popZ*; pSRK-dendra2-zitP^1-43WT(Bd)^ | Transformation of pMB166 into eMB288 |
| eMB338 | TB28; pBAD101- *mCherry -popZ*; pSRK-dendra2-zitP^1-90WT(Ae)^ | Transformation of pMB167 into eMB288 |
| eMB370 | TB28; pBAD101- *mCherry -popZ*; pSRK-*dendra2-zitP*^1-43CS(Cc)^ | Transformation of pMB193 into eMB288 |
| eMB342 | TB28; pBAD101- *mCherry -popZ*; pSRK-*dendra2-zitP*^1-43W35I(Cc)^ | Transformation of pMB169 into eMB288 |
| eMB372 | TB28; pBAD101- *mCherry -popZ*; pSRK-*dendra2-zitP*^1-43R24A/R27A(Cc)^ | Transformation of pMB194 into eMB288 |
| eMB374 | TB28; pBAD101- *mCherry -popZ*; pSRK-*dendra2-zitP*^1-43WT(Rs)^ | Transformation of pMB195 into eMB288 |
| eMB376 | TB28; pBAD101- *mCherry -popZ*; pSRK-*dendra2-zitP*^1-43Q27R(Rs)^ | Transformation of pMB196 into eMB288 |
| eMB378 | TB28; pBAD101- *mCherry -popZ*; pSRK-*dendra2-zitP*^1-43Q27R/W35I(Rs)^ | Transformation of pMB197 into eMB288 |
| eMB311 | TB28; pBAD101- *mCherry -popZ*; pSRK-*dendra2-zitP* | Transformation of pMB43 into eMB288 |
| eMB315 | TB28; pBAD101- *mCherry -popZ*; pSRK-*dendra2-zitP*^1-133WT(Cc)^ | Transformation of pMB86 into eMB288 |
| eMB350 | TB28; pBAD101- *mCherry -popZ*; pSRK-*dendra2-zitP*^1-133W35I(Cc)^ | Transformation of pMB178 into eMB288 |
| eMB352 | TB28; pBAD101- *mCherry* -*popZ*; pSRK-*dendra2-zitP*^1-133malF^ | Transformation of pMB179 into eMB288 |
| eMB330 | TB28; pBAD101- *mCherry* -*popZ* **^Δ26^**; pSRK-*dendra2-zitP*^1-43WT^ | This work |
| eMB132 | MG1655; pSRK-*dendra2*-*zitP*; pBAD101-*popZ* | Transformation of pMB49 into eMB119 |
| eMB153 | MG1655; pBAD101-*popZ* | Transformation of pMB49 into MG1655 |
| eMB380 | MG1655; pBAD101-*popZ*; pSRK-*dendra2*-*zitP* | Transformation of pMB43 into eMB153 |
| eMB382 | TB28; pBAD101*-popZ*; pSRK-*dendra2-zitP*^1-133WT(Cc)^ | Transformation of pMB86 into eMB153 |
| eMB384 | TB28; pBAD101-*popZ*; pSRK-*dendra2-zitP*^1-133W35I(Cc)^ | Transformation of pMB178 into eMB153 |
| eMB386 | TB28; pBAD101-*popZ*; pSRK-*dendra2-zitP*^1-133malF^ | Transformation of pMB179 into eMB153 |
| eMB330 | TB28; pBAD101-*mCherry*-*popZ*^Δ26^; pSRK-*dendra2*-*zitP*^1-43WT^ | Transformation of pMB165 into eMB290 |
| eMB346 | TB28; pBAD101-*mCherry*-*popZ*^Δ26^; pSRK-*dendra2-zitP*^1-133WT^ | Transformation of pMB86 into eMB290 |
| eMB340 | TB28; pBAD101-*mCherry*-*popZ*; pSRK-*dendra2-zitP*^1-43WT(Rm)^ | Transformation of pMB97 into eMB288 |
| eMB346 | TB28; pBAD101-*mCherry*-*popZ*; pSRK-*dendra2-zitP*^1-43W35I(Rm)^ | Transformation of pMB168 into eMB288 |
| eMB388 | TB28; *mcherry-popZ;* pBAD101*-popZ*^(Rm)^; pSRK-*dendra2-zitP*^1-43WT(Cc)^ | Transformation of pMB187 into eMB325 |
| eMB236 | MG1655; pBAD101*-popZ*^(Rm)^; pSRK-*dendra2-zitP*^(Cc)^ | Transformation of pMB107 into eMB119 |
| eMB390 | EC100; pSC-*zitP*^1-43W35I^ | Transformation of into EC100D |
| eMB238 | MG1655; pBAD101- *mCherry -popZ*^(Rm)^; pSRK-*dendra2-zitP*^1-90WT(Cc)^ | Transformation of pMB107 into eMB121 |

| eSC145 | EC100; pSC-*popZ* | | Transformation of pSC-popZ into EC100 |
| --- | --- | --- | --- |
| eSC124 | EC100; pSC-*zitP*^1-43^ | | Transformation of pSC-*2215*^1-43^ into EC100 |
| eMB425 | TB28; pBAD22-*mCherry*-*zitP^1^*^-133^-*popZ* - | | Transformation of pMB224 into TB28 |
| eMB427 | TB28; pBAD22-*mCherry*-*zitP^1^*^-133(W35I)^-*popZ* | | Transformation of pMB225 into TB28 |
| eMB443 | TB28; pBAD22-*mCherry*-*zitP^1^*^-133^-*popZ*; pSRK-*dendra2*-*parB* | | Transformation of pMB233 into eMB425 |
| eMB445 | TB28; pBAD22-*mCherry*-*zitP^1^*^-133(W35I)^-*popZ*; pSRK-*dendra2*-*parB* | | Transformation of pMB233 into eMB427 |
| eMB502 | TB28; pBAD22*-mCherry*-*zitP^1^*^-104(Rm)^-*popZ* | | Transformation of pMB266 into TB28 |
|  |  |  | |

**Plasmids**

| plasmid | description | Source or ref |
| --- | --- | --- |
| pXGFPN-2 | Integration of C-terminal egfp-fusions at Caulobacter P*_xylx_* locus (kanR) | Thanbichler et al., 2007 |
| pXdendra2N2 | Derivative of pXGFPN-2 with dendra2 substituting eGFP | This study |
| pMCS-4 | Vector for chromosomal integration in *C. crescentus* (gentR) | Thanbichler et al., 2007 |
| pNTPS138 | 38-derived integration vector: oriT^+^ sacB^+^ KanR | Alley MRK unpublished |
| pMT335 | High copy plasmid carrying a P_Van_ promoter (gentR) | Thanbichler et al., 2007 |
| pMT464 | High copy plasmid carrying a P_xyl_ promoter (kanR) | Thanbichler et al., 2007 |
| pSRK | pBBR1 broad host range plasmid derived carrying a P_lac_ promoter (kanR) | Khan et al., 2008 |
| pBAD22 | High copy plasmid carrying a P_Bad_ promoter (kanR) | Guzman et al., 1995 |
| pBAD101 | Low copy plasmid carrying a P_Bad_ promoter (spcR) | Bieler et al., 2006 |
| pSC | Derivative of pET26b | This study |
| pMB123 | pNTPS138-Δ*zitP* | This study |
| pXdendra2-*zitP* | pXdendra2N-2-*zitP* | This study |
| pMB22 | pXdendra2N-2-*zitP*^1-90WT(Cc)^ | This study |
| pMB33 | pXdendra2N-2-*zitP*^1-90WT(Ae)^ | This study |
| pMB38 | pXdendra2N-2-*zitP*^1-90WT(Mm)^ | This study |
| pMB39 | pXdendra2N-2-*zitP*^1-90WT(Cs)^ | This study |
| pMB66 | pXdendra2N-2-*zitP*^1-43WT(Cc)^ | This study |
| pMB73 | pXdendra2N-2-*zitP*^1-43WT(Bd)^ | This study |
| pMB26 | pXdendra2N-2-*agmX*^1-90WT^ | This study |
| pMB21 | pMT335-*popZ* | This study |
| pMB165 | pSRK-*dendra2*-*zitP*^1-43WT(Cc)^ | This study |
| pMB166 | pSRK-*dendra2*-*zitP*^1-43WT(Bd)^ | This study |
| pMB167 | pSRK-*dendra2*-*zitP*^1-90WT(Ae)^ | This study |
| pMB164 | pXdendra2N-2-*zitP*^1-43CS(Cc)^ | This study |
| pMB115 | pXdendra2N-2-*zitP*^1-43W35I(Cc)^ | This study |
| pMB170 | pXdendra2N-2-*zitP*^1-43R24A/R27A(Cc)^ | This study |
| pMB193 | pSRK-*dendra2*-*zitP*^1-43CS(Cc)^ | This study |
| pMB169 | pSRK-*dendra2*-*zitP*^1-43W35I(Cc)^ | This study |
| pMB194 | pSRK-*dendra2*-2-*zitP*^1-43R24A/R27A(Cc)^ | This study |
| pMB184 | pXdendra2N-2-*zitP*^1-43WT(Rs)^ | This study |
| pMB91 | pXdendra2N-2-*zitP*^1-43Q27R(Rs)^ | This study |
| pMB185 | pXdendra2N-2-*zitP*^1-43Q27R/W35I(Rs)^ | This study |
| pMB195 | pSRK-*dendra2*-*zitP*^1-43WT(Rs)^ | This study |
| pMB196 | pSRK-*dendra2*-*zitP*^1-43Q27R(Rs)^ | This study |
| pMB197 | pSRK-*dendra2*-*zitP*^1-43Q27R/W35I(Rs)^ | This study |
| pMB43 | pSRK-*dendra2*-*zitP* | This study |
| pMB86 | pSRK-*dendra2*-*zitP*^1-133WT(Cc)^ | This study |
| pMB178 | pSRK-*dendra2*-*zitP*^1-133W35I(Cc)^ | This study |
| pMB179 | pSRK-*dendra2*-*zitP*^1-133malF^ | This study |
| pMB49 | pBAD101-*popZ* | This study |
| pMB59 | pBAD101- *m****C****herry* -*popZ* | This study |
| pMB175  pMB177  pMB93  pMB94  pMB113  pMB171  pMB127  pMB156 | pMT464-*zitP*^1-133WT(Cc)^-*popZ*  pMT464-*zitP*^1-133W35I(Cc)^-*popZ*  pMT464-*zitP*^1-133WT(Cc)^  pMT464-*zitP*^1-133CS(Cc)^  pMT464-*zitP*^1-133W35I(Cc)^  pMT464-*zitP*^1-133R24A/R27A(Cc)^  pMT464-*zitP*^1-133malF(Cc)^  pMT464-*zitP*^1-133WT(Bd)^ | This study  This study  This study  This study  This study  This study  This study  This study  This study |
| pMB23 | pXdendra2N-2-*zitP*^1-133WT(Cc)^ |  |
| pMB25 | pXdendra2N-2-*zitP*^1-133CS(Cc)^ | This study |
| pMB145 | pMCS-4- *m****C****herry* -*popZ*^Δ26^ | This study |
| pMB128 | pBAD101- *m****C****herry* -*popZ*^Δ26^ | This study |
| pMB81 | pXdendra2N-2-*zitP*^1-43WT(Rm)^ | This study |
| pMB116 | pXdendra2N-2-*zitP*^1-43W35I(Rm)^ | This study |
| pMB97 | pSRK-*dendra2*-*zitP*^1-43WT(Rm)^ | This study |
| pMB168 | pSRK-*dendra2*-*zitP*^1-43W35I(Rm)^ | This study |
| pMB114 | pMT464-*zitP*^1-104WT(Rm)^ | This study |
| pMB181 | pSRK-*dendra2*-*zitP*^1-120WT(Rm)^ | This study |
| pMB187 | pBAD101- *m****C****herry* -*popZ*^(Rm)^ | This study |
| pMB107 | pBAD101-*popZ*^(Rm)^ | This study |
| pMB64 | pSC-*popZ* | This study |
| pMB140 | pSC-*zitP*^1-43W35I^ | This study |
| pSC-zitP^1-43WT^ | pSC-*zitP*^1-43^ | This study |
| pMB233 | pSRK-*dendra2-parB* | This study |
| pMB267 | pBAD22-*mCherry*-*popZ* | This study |
| pMB224 | pBAD22-*mCherry*-*zitP^1^*^-133^-*popZ* | This study |
| pMB225 | pBAD22-*mCherry*-*zitP^1^*^-133(W35I)^-*popZ* | This study |
| pMB255 | pMT464-*zitP^1^*^-90^ | This study |
| pMB256 | pMT464-*zitP* | This study |
| pMB257 | pMT464-*zitP*^1-90^ -*popZ* | This study |
| pMB258 | pMT464-*zitP*-*popZ* | This study |
| pMB265 | pMT464-*zitP^1^*^-104(Rm)^-*popZ* | This study |
| pMB266 | pBAD22-*mCherry*-*zitP^1^*^-104(Rm)^-*popZ* | This study |

**Oligonucleotides**

| name | sequence |
| --- | --- |
| Van | 5’- GCCGACCGACTGAGACGCTCACAA -3’ |
| T7pro | 5’- AAATTAATACGACTCACTATA -3’ |
| Xyl | 5’- AACCTACTTGCCGTCCCCACA -3’ |
| Lac290 | 5’- tgacggctatcaccatcat -3’ |
| T7ter | 5’- TCAAGACCGGTTTAGAGGCCCCAA -3’ |
| xylAS | 5’- TTGTCTGACCAGCGCGAAATCCT -3’ |
| M13(-20) | 5’- GCCAGGGTTTTCCCAGTCACGA -3’ |
| 2215sh_ndeI | 5’- AAAAAAcatatgATACTGACCTGCCCGGA -3’ |
| 2215shCS_NdeI | 5’-AAAAAACATATGATACTGACCTCCCCGGA-3’ |
| CC2215_E | 5’-AAAAAAGAATTCATTCATGATGAGCGGGTTCGTGAGA-3’ |
| 2215shCS+2_SacI | 5’-AAAAAAGAGCTCTAATGATACTGACCTCCCCGGA-3’ |
| dendra2N_F | 5’-ACGACCATATGAACACCCCGGGAATTAACC-3’ |
| dendra2N _R | 5’-GCATATTAATTAAGGCGCCTGCAGGCCACACCTGGCTGGGCAGG-3 |
| zitP_Cterm_nde | 5’-AAAAAACATATGCTGCTGCCGACCGTCAGCCTGATCGA-3’ |
| zitP_Cterm_eco | 5’-AAAAAAGAATTCAGGCCGCCCGAATCCCCGAGCCGCTCT-3’ |
| OMB87 | 5’-AAAAAgaattcAGGGTCGGACGCGCGGCGCGCGCTACAA -3’ |
| OMB88 | 5’- AAAAggattcGCAGGTCAGTATCATGGCCGCGAATCGAA -3’ |
| OMB89 | 5’- AAAAAAggattcCAGGAACCCCCGGGACATGAGA -3’ |
| OMB90 | 5’- AAAAAAaagcttGGCGCGGCCATGGCCAGCGAGTACAAT -3’ |
| OMB12 | 5’- AAAAAAgagctctaatgatactgacc -3’ |
| OMB13 | 5’- AAAAAAgaattctcacttgggaagttcttcg -3’ |
| OMB41 | 5’- AAAAAgaattcATTCAGCTTCGTCCTTG -3’ |
| OMB14 | 5’- AAAAAAgaattctcaatcgatccggaagatc -3’ |
| OMB13 | 5’ AAAAAAgaattctcacttgggaagttcttcg -3’ |
| OMB7 | 5’- aaaaacatatgtccgatcagtctcaagaa -3’ |
| OMB11 | 5’- AAAAAAgaattcAGGCGCCGCGTCCCCGAGAGA -3’ |
| OMB70 | 5’- AAAAAggtaccCCGGCGGAGCCTGCGG -3’ |
| OMB35 | 5’- AAAAAccatggggatccacggtggtggtggtg -3’ |
| OMB36 | 5’- AAAAAcatatgTCCGGAACCcttgtagagctcatccatgccg -3’ |
| OMB65 | 5’- AAAAAcatatgCCGGCGGAGCCTGCGG -3’ |
| OMB66 | 5’- AAAAAtctagatcaggcgccgcgtccccgag -3’ |
| OMB76 | 5’- AAAAAgtgaagctgtacgagcacgcc -3’ |
| OMB80 | 5’- AAAAAgagctctaATGATCCTGACGTGCCCCG -3’ |
| OMB81 | 5’- AAAAAgagctctaATGATTCTGACCTGTCCGG -3’ |
| OMB83 | 5’- AAAAAgagctctaatgTACATCACCTGTCCGAAC -3’ |
| OMB84 | 5’- caccgcggtggcg -3’ |
| OMB18 | 5’- AAAAAAcatatgaacaccccgggaattaac -3’ |
| OMB134 | 5’- AAAAAggatccCCACACCTGGCTGGGC -3’ |
| OMB130 | 5’- AAAAAggatccATGGAGTCCGTCGTG -3’ |
| OMB131 | 5’- AAAAAaagcttTCAGATCCCGCGCGTC -3’ |
| zitP1-43_fw_NdeI | 5’- ATATACATATGATCCTGACTTGTCCGG-3’ |
| zitP1-43_bw_XhoI | 5’- ATATCTCGAGTTCAGCTTCATCTTTGAATGCC-3’ |
